# Site-specific and global glycan analysis of the human cytomegalovirus pentameric glycoprotein by high resolution mass spectrometry

**DOI:** 10.1093/glycob/cwag062

**Published:** 2026-08-05

**Authors:** Antonio Lembo, Gian Luca Sardone, Michela Aurilia, Immacolata Speciale, Cristina De Castro, Massimiliano Biagini

**Affiliations:** Department of Chemical Sciences, University of Naples Federico II, Via Vicinale Cupa Cintia, 26, 80126 Naples, Italy; GSK Vaccines, Analytical R&D, Via Fiorentina, 1, 53100, Siena, Italy; GSK Vaccines, Analytical R&D, Via Fiorentina, 1, 53100, Siena, Italy; GSK Vaccines, Analytical R&D, Via Fiorentina, 1, 53100, Siena, Italy; Department of Chemical Sciences, University of Naples Federico II, Via Vicinale Cupa Cintia, 26, 80126 Naples, Italy; Department of Chemical Sciences, University of Naples Federico II, Via Vicinale Cupa Cintia, 26, 80126 Naples, Italy; GSK Vaccines, Analytical R&D, Via Fiorentina, 1, 53100, Siena, Italy

**Keywords:** cytomegalovirus, glycans, glycoprotein, mass spectrometry, vaccines

## Abstract

The Human Cytomegalovirus (HCMV) gH/gL/UL128/UL130/UL131A complex (Pentamer) is among the candidates for the development of a vaccine against HCMV; indeed, it brought to more immunogenic formulations which were able to protect the fetus against vertical transmission in pregnant women. Pentamer-specific antibodies have shown to be hundredfold more potent than gB or gH/gL antibodies, endowed with strong neutralizing activity. The often inadequate antibody immune response elicited by glycoprotein antigen has been, in part, attributed to the abundance of glycosylation on the protein. Here, we determine the composite glycan population of each of the N-linked and O-linked glycosylation sites present on the Pentamer by multienzymatic proteolysis and mass spectrometry; furthermore, total N-glycans profiles of Pentamer expressed in different cell systems have been compared exploiting the glycan fluorescent labeling and the immunogenicity of Pentamer with different glycosylation patterns has been investigated. Our analysis reveals the presence of underprocessed oligomannose-type glycans on some glycosylation sequons, which are probably derived as a result of sterically reduced accessibility to glycan processing enzymes. Furthermore, the antigen glycosylation pattern affects the elicitation of neutralizing antibodies in mice.

## Introduction

Human Cytomegalovirus (HCMV) is one of the eight herpesviruses known to infect humans, it is perhaps the most ubiquitous of human infections. Indeed, virtually 100% of adults in low- and middle-income countries have been infected when young (Plotkin and Boppana 2019). The infection by this virus is asymptomatic in immunocompetent people, however it can be very dangerous in immunocompromised patients (AIDS patients, transplant patients) and in infants in utero, causing pneumonia, retinitis and life-long neurological damages (Griffiths et al. 2015). The rate of congenital HCMV transmission is between 0.5% and 0.7% of pregnancies in developed nations, and up to 2% of pregnancies in the developing world (Scarpini et al. 2021). Furthermore, before the development of antiretroviral therapy, up to 40% of AIDS patients had severe HCMV disease. Currently, the development of a vaccine against HCMV is still in progress; among promising candidate antigens there is the pentameric complex gH/gL/UL128/UL130/UL131A (or Pentamer), which is a HCMV surface glycoprotein. This complex is important for eliciting protective humoral immune response and is the predominant target of neutralizing antibodies in humans, accounting for >85% neutralizing activities in natural infection. Moreover, pentameric complex is associated with protection against transmission to the fetus (Macagno et al. 2010), (Plotkin and Boppana 2019).

The dimeric gH/gL complex is a covalent complex, where gL-Cys^47^ and gL-Cys^54^ form two disulfide bonds with gH-Cys^95^ and gH-Cys^59^ respectively; it can interact either with gO or with the ULs proteins (UL128, UL130, UL131A) to form gH/gL/gO (trimer) and gH/gL/UL128/UL130/UL131A (pentamer) respectively.

The type of complex that will be assembled will define the cell tropism (kind of cell that virus can infect). In fact, HCMV entry into fibroblasts is mediated by gH/gL/gO (recent data suggest that it may contribute to entry into all cell types), while HCMV entry into dendritic, epithelial, endothelial cells and leukocytes requires the pentameric glycoprotein complex (Zhou et al. 2015), (Chandramouli et al. 2017).

**Figure 1:**
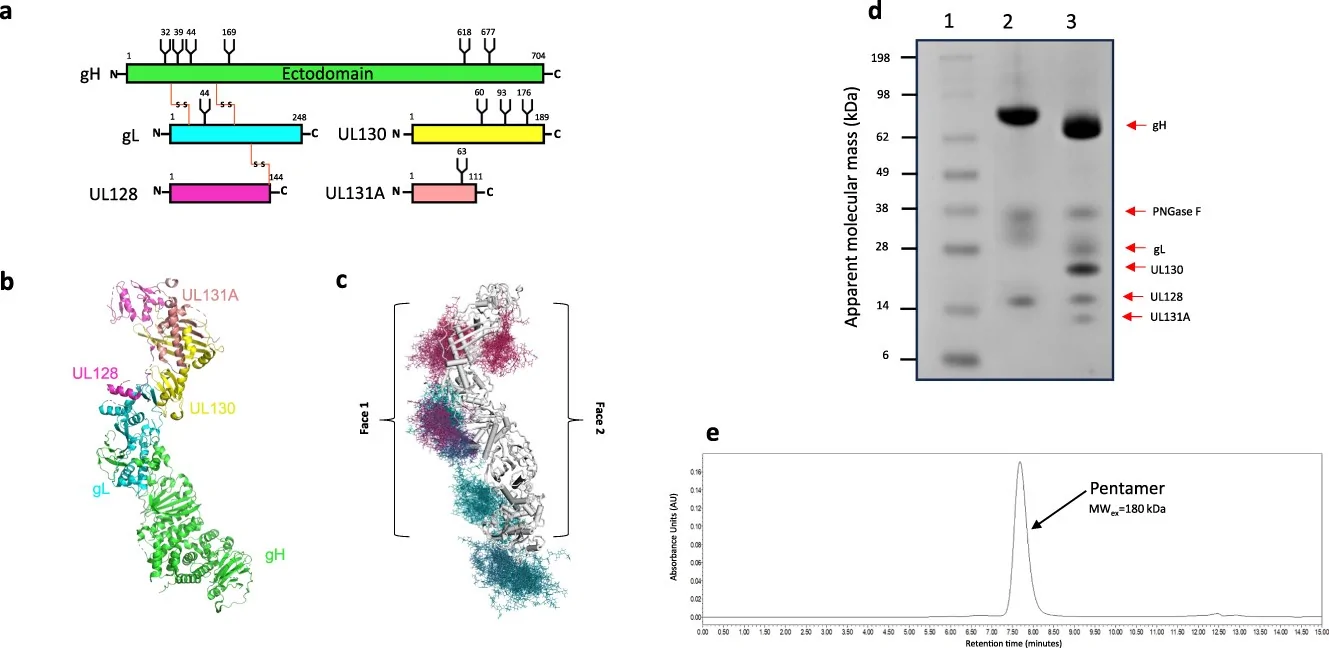
a) Schematic representation of HCMV pentamer. The positions of N-linked glycosylation sequons (N-X-S/T, where X ≠ P) are shown as branches. b) Crystal structure of pentamer (PDB code 5VOB). c) Pentamer faces decorated with 30 N-glycan conformers (M5 structures) for each N-glycosylation site by GlycoSHIELD (Tsai et al. 2024); red colored conformers play a more localized shielding effect of the protein backbone due to their lower conformational freedom. d) Denaturing gel electrophoresis of pentameric complex. Lane 1: SeeBlue Plus2 pre-stained protein standard. Lane 2: Pentameric complex. Lane 3: Pentameric complex after PNGase F treatment. Proteins were separated onto a NuPAGE 4%–12% bis-tris polyacrilamide gel and stained by Coomassie-blue. e) SEC-UV profile of the affinity-purified pentamer from CHO cells.

Pentamer is a complex with an apparent molecular mass of ≈180 kDa formed by 5 different proteins (gH/gL/UL128/UL130/UL-131A) (Fig. 1a), where the gH/gL dimer is covalently linked to UL128 through a disulfide bond (between UL128-Cys^144^ and gL-Cys^162^) and non-covalently with UL130 and UL131A, with the function of defining cell tropism towards endothelial and epithelial cells (Ciferri et al. 2015a). It adopts a helicoidal shape of 180 Å in length and 30 to 80 Å in cross section; the gH/gL part of the complex has a similar domain organization to that of other herpesvirus, with gH domains (D-I to D-IV) stacking on top of each other. The polypeptide chains of the ULs form a gently curved subcomplex that binds to an extension at the N-terminus of gL (Fig. 1b). The pentameric complex recognizes neuropilin 2 (NRP2, a human receptor involved in semaphorin and vascular endothelial growth factor, VEGF, binding) and this interaction triggers the transition of the viral fusogen protein gB from its pre- to post-fusion conformation, initiating membrane fusion; this pathway is principally adopted for epithelial cells infection (Kschonsak et al. 2022), (Martinez-Martin et al. 2018).

The pentameric complex has 11 N-glycosylation sequons: 6 in gH, 1 in gL, 3 in UL130 and 1 in UL131A (Chandramouli et al. 2017) (Fig. 1a); the different number of glycosylation sites own by each monomer affects their electrophoretic migration. In the case of UL128 (which has no N-glycosylation sequons) and UL131A (which has 3 N-glycosylation sequons), we can see that before PNGase F treatment they migrate on electrophoretic gel at the same speed (only one band seen), while after N-glycans shaving the two proteins are resolved (Fig. 1d); indeed, without N-glycan UL131A has a greater mass difference with UL128, and its migration is not hampered by the bulky oligosaccharide anymore. To assess that in the native form all the glycoprotein subunits are associated, a SEC (Size Exclusion Chromatography)-UV analysis has been performed, which has shown only one peak at the expected retention time, with a measured molecular weight of 180 kDa (Fig. 1e), protein contribute is 160 kDa, the remaining weight is from glycans. The pentameric complex is characterized by two different faces, one decorated with 10 glycans (Fig. 1c, Face 1) while the other face features a single glycan in UL130 and positively charged areas with clusters of exposed arginine and lysine residues (Fig. 1c, Face 2), (Chandramouli et al. 2017). Glycans analysis is a powerful tool to understand in depth which are the most shielded protein regions by the oligosaccharides, this can address researchers towards the development of more immunogenic glycoproteins with tailor-made glycan patterns (Lembo et al. 2026), (Watanabe et al. 2020) and further clarify receptor binding mechanism (Paiardi et al. 2024). Moreover, the nature of glycoforms which decorate the antigen surface can affect both half-life and safety of the product (Rocamora et al. 2023).

Hitherto, the glycan pattern of the Pentameric complex from HCMV strain Merlin has not been characterized. Here we illustrate the use of high-resolution LC–MS technology to analyze in a semi-quantitative fashion the surface glycans of this glycoprotein complex; moreover, glycan patterns and immunogenicity of Pentamer from different expression conditions have been compared.

## Results

### N-glycans site-specific analysis

#### Microheterogeneity analysis

N-glycans site-specific microheterogeneity analysis was performed using a proteomic bottom-up approach on glycoprotein expressed in CHO cells. After denaturation, Pentamer glycopeptides were generated by digestion with different proteases (trypsin, chymotrypsin, and α-lytic protease) and subjected to in-line liquid chromatography–tandem mass spectrometry (LC–MS/MS). The use of different proteases allowed to cover all the peptide sequences containing N-glycosylation sequons. Glycopeptides for relative quantification were selected according to specific physico-chemical features: minimum number of consensus sequences (ideally only one), specific cleavage by the used protease, presence of oxonium ions from glycan fragmentation (at least two) and good primary sequence coverage (both *b* and *y* ion series must be detected in the MS/MS fragmentation spectrum). The latter is reached using the function *“glycan search”* of PEAKS Studio 11 software, which allowed us to obtain a full characterization of the glycopeptides MS/MS spectra, accounting also for *b/y* ion series which bring variable glycan fragments to the peptide backbone (Sun et al. 2023) (Fig. S1).

Site-specific glycans relative quantitation was obtained by summing the ion intensity of the corresponding glycopeptide over all identified charge states, using the corresponding extracted area (XICs), and dividing it by the total intensity of all the glycopeptide peaks present for a specific site (label free approach). Protonation occurs on the peptide portion, and thus the signal intensity of the glycopeptide is largely dependent on the proton affinity of peptide component of the molecule, thus rationalizing the quantitation of different glycoforms of a peptide according to their signal intensities (Wada et al. 2007; Behrens et al. 2016).

**Figure 2:**
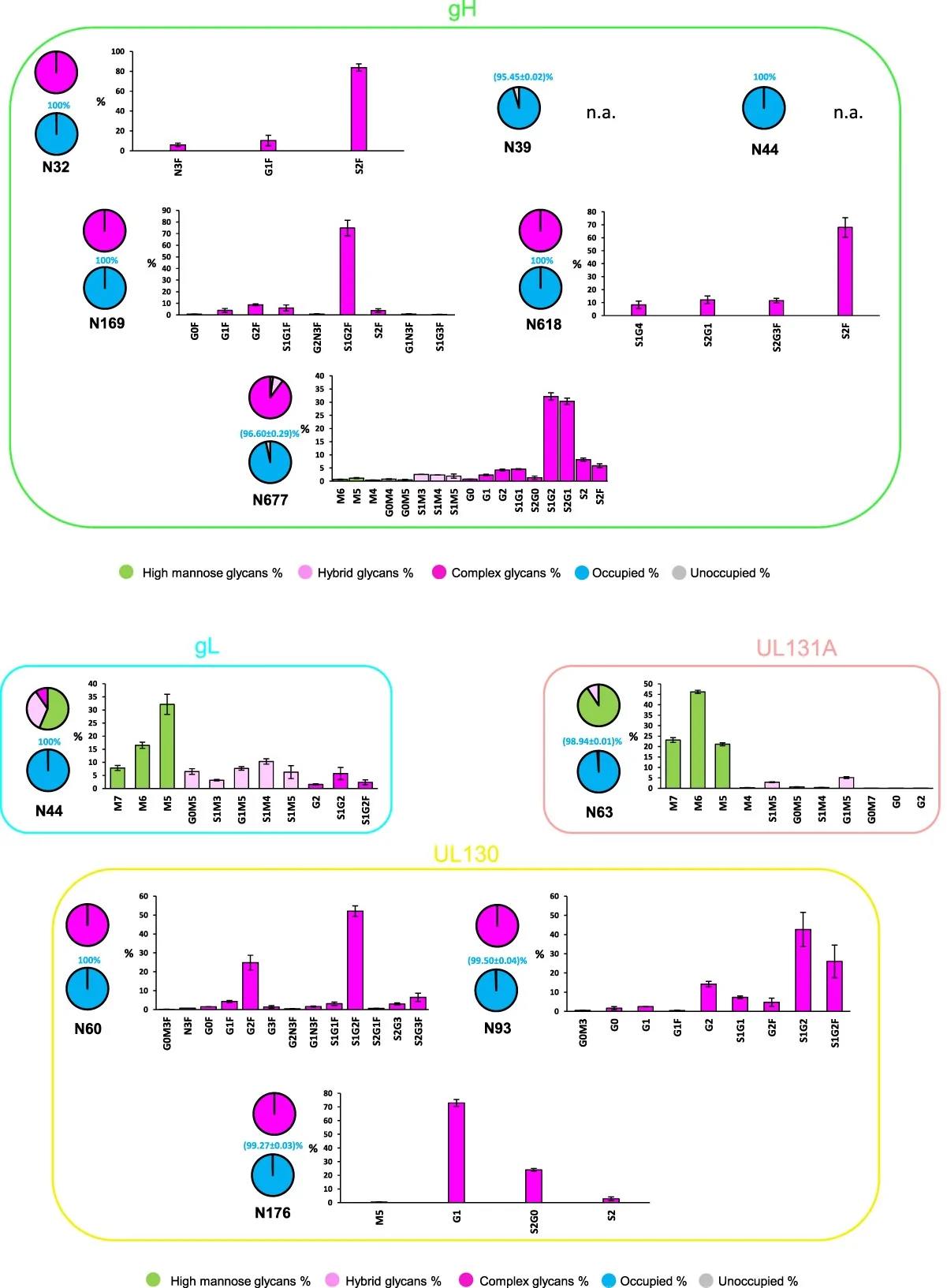
Site-specific N-glycans relative abundances and occupancies on HCMV pentamer (more details in Supplementary Tables 1, 2). N-glycan structures are reported in Fig. S2. Bars indicate the associated analytical errors (± standard deviation). The acceptability criteria used to define the limit of quantitation (LOQ) are relative standard deviation (RSD) ≤ 55% and signal/noise ≥10.

This analysis revealed that each N-glycosylation site presents a diverse range of glycan compositions. The most abundant complex glycan is the structure S1G2F, which has been found on almost half of the analyzed glycosylation sites (UL130 N60 and N93; gH N169, gL N44); otherwise, biantennary complex glycans (S2F) and non-sialylated glycans (G1) have been found (Fig. 2). Unusual structures S2G1 and S2G0 (which exhibits a Neu5Ac-GlcNAc bond) have been found on sites N618, N677 (gH) and N60 (UL130), their attribution has been confirmed by the presence of oxonium ions at *m/z* 292.10, 366.14, 528.19 and 657.24 for the first one, and at *m/z* 292.10, 366.14, 657.24 for the second one (Fig. S1).

**Figure 3:**
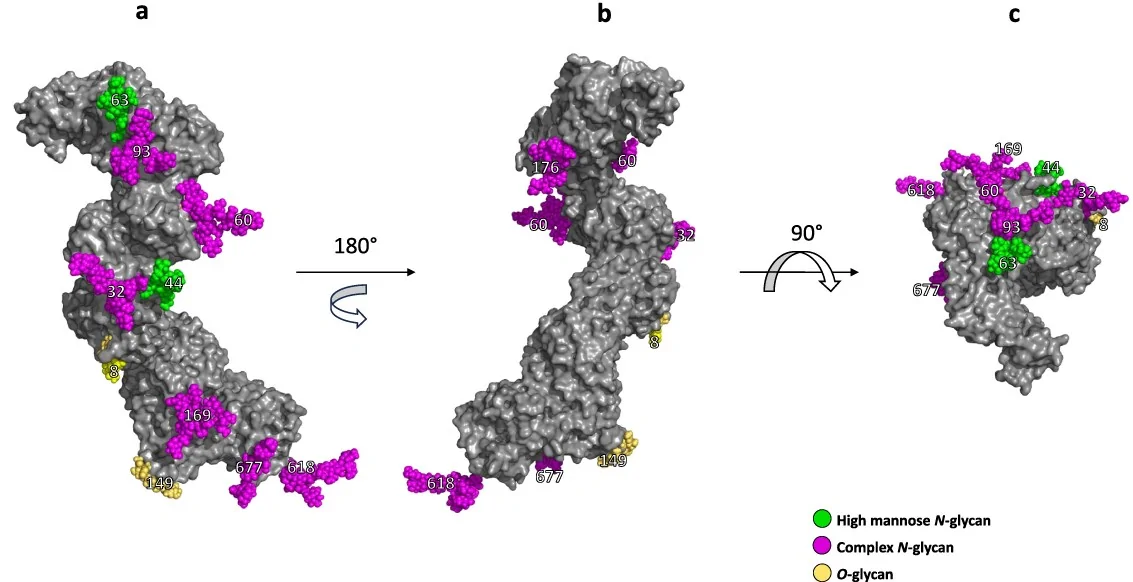
The glycosylated model of pentamer was constructed using the PDB code 5VOD and GlycoSHIELD (Tsai et al. 2024). The pentamer protein backbone is shown as a grey surface and the glycans are shown as spheres of different colors depending on the most abundant glycoform experimentally detected at that site. a) Side view of the model. b) Side view of the model. c) Top view of the model. Sites N39(gH), and N44(gH) have not been reported, S4(gL) is not present in the crystal (9/11 N-glycosylation sites and 2/3 O-glycosylation sites are visualized in the model derived from crystal coordinates).

We observed that only two sites (N44 gL and N63 UL131A) were predominantly composed of oligomannose type (Fig. 2). Sequon N44 on gL is located on a flexible loop at the center of the groove formed by gH, gL and UL130 (Fig. 3a); the oligosaccharide on this site is surrounded by two other glycans (N32 from gH and N60 from UL130) (Fig. 3a). The steric hindrance played by the two lateral glycan structures hampers processing enzymes accessibility to glycan N44, giving rise to the oligomannose structures M7, M6 and M5 (Fig. 2). Glycosylation site N63 on UL131A is the most underprocessed one, with a high mannose structures percentage of 90% (Fig. 2). This consensus is sited near the contact region between UL128 and UL131A, which partially shields the glycan from the action of glycosidases, moreover, the N63 glycan is wrapped up by the helicoidal protein backbone and the most external N93 glycan (Fig. 3c).

Despite occupied at nearly 100%, sites N39 and N44 on gH did not show glycoforms according to the analytical method used; nevertheless, the presence of glycan structures on these sites has been further confirmed by glycopeptide mapping experiments after N-glycans trimming of the denatured glycoprotein with the glycosidases EndoH and EndoF3, which selectively cleave different types of N-glycan structures within the chitobiose core, leaving a single GlcNAc residue attached to the asparagine. The trimming of the glycans before digestion increases protease accessibility to cleavage sites, and reduces the polarity of the peptides, preventing the analyte from getting stuck on the SPE column and improving ionization efficiency, allowing to detect them by MS. In the case of sites N39 and N44, trypsin did not succeed in separating these amino acids on two different glycopeptides, because of the glycan hindrance towards R43, giving a glycopeptide bearing two oligosaccharides, which are known to lower in a significative way the ionization efficiency of the analyte, that in this case is not seen (Stavenhagen et al. 2013).

#### N-glycosylation site occupancy analysis

N-glycosylation site occupancy (known also as macroheterogeneity) refers to the stoichiometry of substitution of a certain glycosylation site. These differences in glycosylation level are dependent on the manufacturing system (e.g. organism/cell-type-specific) and correlated to protein intrinsic features.

After digestion and purification, Pentamer glycopeptides were dried and treated with the enzyme PNGase F in H_2_^18^O before injection into the LC–MS/MS system; this approach gives a more accurate occupancy measurement because the isotopic labelling can distinguish between enzymatically deamidated peptides from non-enzymatically deamidated peptides; indeed, non-enzymatic deamidation of Asn and Gln occurs spontaneously on proteins and peptides both in vivo and in vitro; the deamidation rate increases significantly under alkaline conditions and at relatively high temperature (Hao et al. 2011; Pace et al. 2013). Therefore, slightly acid pH and reduced heating times have been used during protein denaturation to minimize this effect. Glycan removal from glycosylation site before occupancy analysis allows to bypass the issues related to different ionization efficiency of glycopeptides compared to nonglycosylated peptides, indeed, the former have weaker ionization than the latter (Rebecchi et al. 2009).

For occupied sites, the enzymatic reaction will bring to peptides with a + 2.9840 Da mass shift, while for peptides with unoccupied glycosites these treatments produce no mass shift or a mass shift of +0.9840 Da if deamidation has occurred (Cao et al. 2017). Furthermore, tryptic incorporation of one or two ^18^O into the C-terminus of peptides during N-linked glycosylation site mapping has been considered during peptide mapping, indeed this modification can lead to incorrect and ambiguous identification of sites of N-linked glycosylation; this issue has been overcome by allowing ^18^O incorporation into the C-terminus as a variable modification during the database search (+2.0042 Da or + 4.0082 Da) (Angel et al. 2007) (Fig. S3).

The analysis outputs showed that all the Pentamer N-glycosylation sites displayed occupancy values near or equal to 100% (pie chart in Fig. 2). The measurements have been made by extracting and integrating the ion chromatogram of the labeled glycopeptide (considering all the charges states) and dividing the area by the total area, obtained summing the area of the labeled and not labeled portions. Sites that did not show any unlabeled peptide have been considered 100% occupied.

To assess the shielding that glycans play on Pentamer surface, a tridimensional model of the oligosaccharides on the protein has been built using GlycoSHIELD (Tsai et al. 2024); indeed, glycans are much more dynamic than folded polypeptides, exploring extensive arrays of conformations over tens of nanoseconds, hence creating molecular shields that mask large swaths of protein surface (Fig. 3).

### Released *N*-glycans analysis

Total N-glycans analysis of Pentamer expressed in different expression cells (CHO and Expi293 GnT1- cells), has been performed by liquid chromatography-fluorescence-tandem mass spectrometry (LC-FLR-MS/MS) system. The rationale behind expression systems selection is that CHO cells have shown in previous studies to express the pentamer in a native and soluble form, with epitopes and stoichiometry identical to those found on the protein originating from the virus (Loughney et al. 2015); on the opposite, the expression of correctly folded mutant forms of the pentamer has been successfully achieved previously in human cells such as Expi293 cells or HEK cells (Chandramouli et al. 2017). Thus, both the expression systems have proven to provide the same protein construct in which the only variable appears to be the N-glycosylation pattern.

N-glycans shaving on Pentamer has been performed using PNGase F, then the enzymatically released N-glycans have been labeled with Rapifluor-MS, a both fluorescent and ionizable tag; the derivatization allows to make glycans detectable by fluorescence, giving a higher response than mass spectrometer detection, in fact, glycans are poor ionizable and the ionization relies on the type of glycan. In addition, RapiFluor-MS normalizes the signal of each oligosaccharide, because the fluorescence is owed to the tag quinoline portion; furthermore, the ionizable RapiFluor-MS amino group improves the glycan detection by mass spectrometry, which is useful for high accuracy glycan identification, especially in the case of complex mixtures in which glycans are not fully resolved by chromatography. Glycans have been relatively quantified using the fluorescence signal, while their identities have been assigned using the total ion chromatograms (TICs) obtained by high resolution mass spectrometry (Deris et al. 2021).

**Figure 4:**
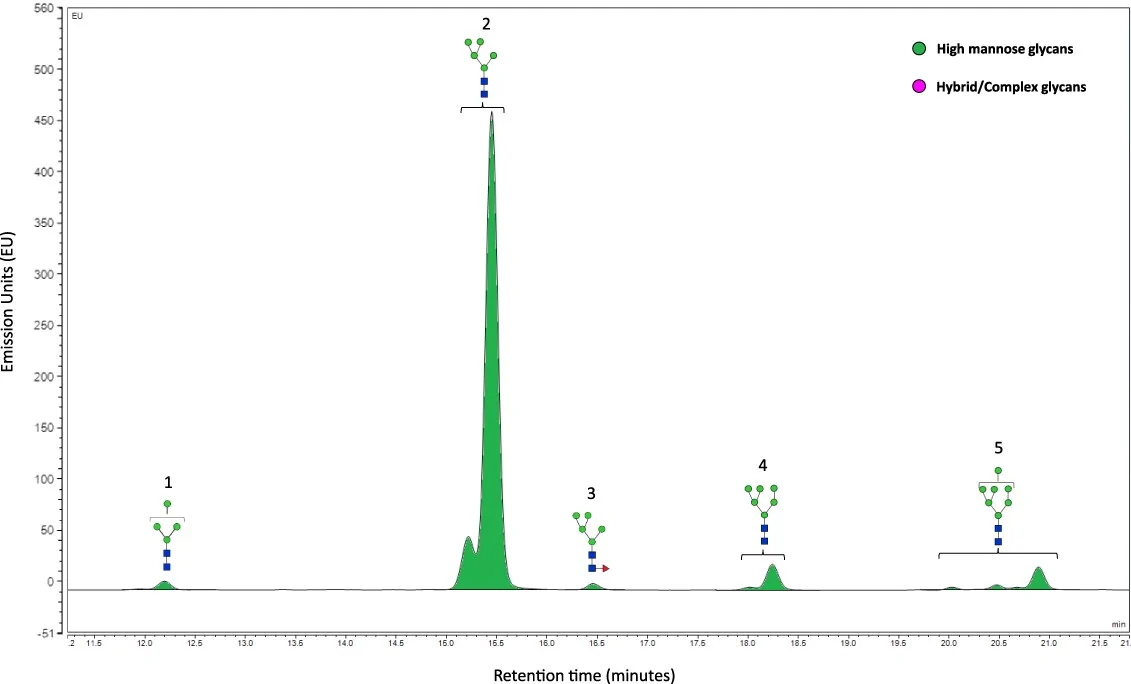
LC-fluorescence profile of released N-glycans from pentamer expressed in Expi293 GnT1- cells (more details in Supplementary Table 3). Monosaccharide symbols follow the SNFG (symbol nomenclature for glycans) system (Varki et al. 2015).

The N-glycans pattern of Pentamer expressed with Expi293 GnT1- has shown only a predominant structure, which is the high mannose glycan M5 (Fig. 4, peak 2); in this case the glycan maturation is arrested to the N-acetylglucosaminyltransferase I (GnT1) level. Lower levels of other high mannose structures like M4, M5F, M6 and M7 have been detected. No complex structures have been detected (Fig. 4).

**Figure 5:**
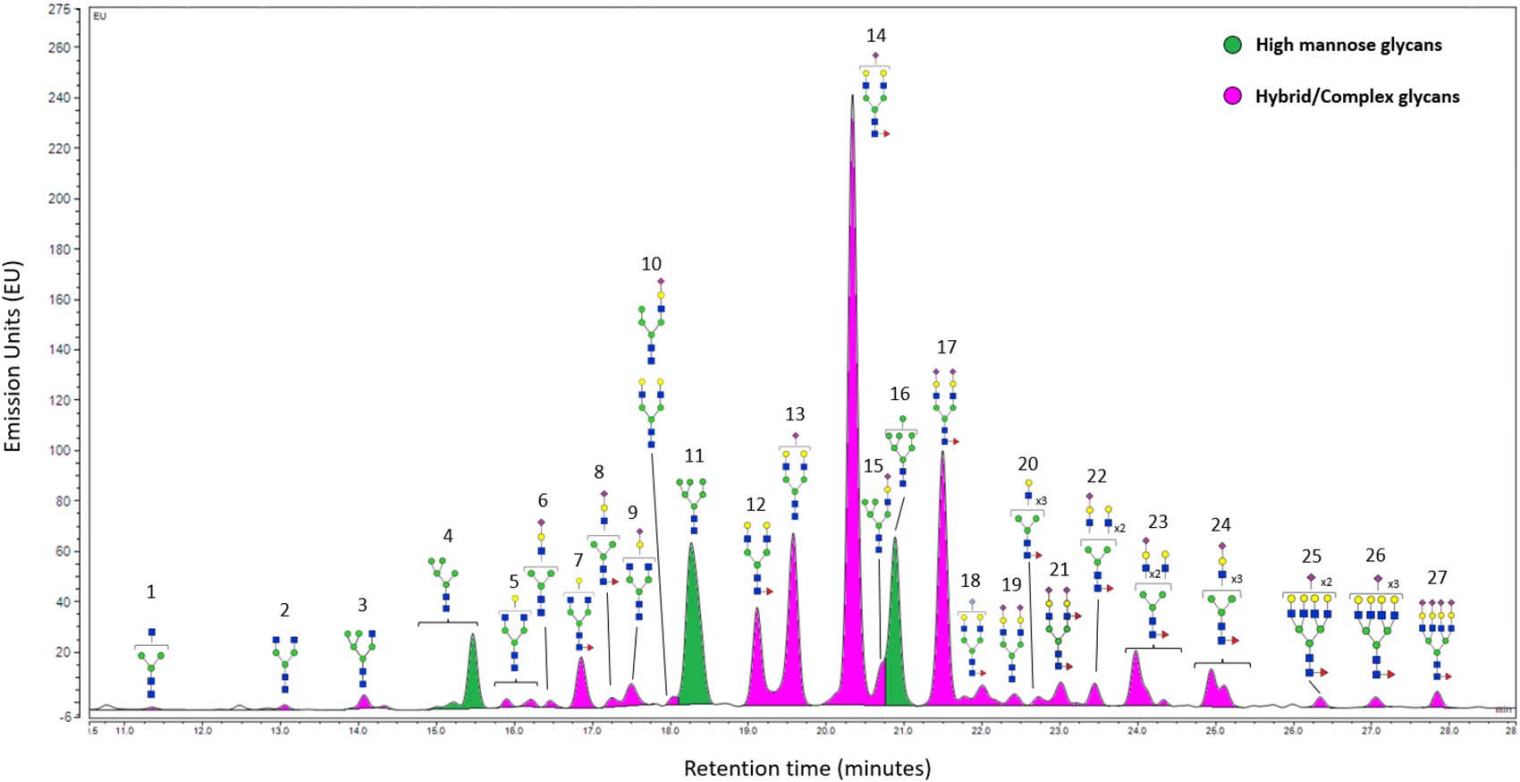
LC-fluorescence profile of released N-glycans from pentamer expressed in CHO cells (more details in Supplementary Table 4). Monosaccharide symbols follow the SNFG (symbol nomenclature for glycans) system (Varki et al. 2015).

The N-glycans pattern of Pentamer expressed in CHO cells has shown two predominant structures, which are the sialylated complex glycans S1G2F and S2F (Fig. 5, peak 14 and 17), meaning that glycans have high maturation degree; however, a lower but significant percentage of high mannose glycans M5, M6 and M7 has been detected (Fig. 5, peak 4, 11, 16). Glycan structures containing Neu5Gc have been detected with a very low relative abundance, indeed, their intensities were comparable with the background noise; the confirmation of the presence of Neu5Gc has been done by detecting the *m/z* 308.09761 (Neu5Gc) and *m/z* 290.08759 (Neu5Gc-H_2_O) oxonium ions in the glycans MS/MS spectra (Fig. S4). Consistent with the LC–MS/MS site-specific analysis, we observed that most of the glycans were of the complex type, with structure S1G2F as most abundant. The presence of a sLeX antigen containing glycan (S2F2) has been detected in low quantities and supported by the presence of oxonium ions at *m/z* 350.14 and 803.29 (Fig. S4), this species is generally not biosynthesized in wild-type CHO cells.

### O-glycans site-specific analysis

O-glycans of viral glycoproteins appear in two types of patterns: single O-glycans scattered along the amino acid sequence of certain viral glycoproteins and multiple O-glycans densely packed in clusters, called mucin-like domains (MLDs). It has been shown that O-glycans on some viral glycoproteins (like HSV-1 gC) are necessary not only for adjusting viral binding to their initial receptor (e.g. heparane sulphate) but also for preventing progeny virus from entrapment on the dying cell in which the virus replicates (Altgarde et al. 2015). While some O-glycosylation sites are universal, others show tissue-dependent combinations of different Ser/Thr sites (Olofsson et al. 2023). However, it has been noted that regions of peptides which contain a high proportion of Ser, Thr and Pro residues are frequently O-glycosylated (Hema Thanka Christlet and Veluraja 2001).

A bottom-up glycoproteomic approach was used to study Pentamer O-glycosylation in CHO cells; after denaturation, Pentamer glycopeptides were generated by digestion with either trypsin or chymotrypsin and subjected to in-line LC–MS/MS. As well as N-glycopeptides, O-glycopeptides were selected according to specific physico-chemical features: specific cleavage, presence of peptide ions bringing the intact glycan portion and good primary sequence coverage (both *c* and *z* ion series must be detected, using ETD fragmentation). The O-glycosylation mapping has been performed exploiting the function *“glycan search”* of PEAKS Studio 11 software, which allowed a full characterization of the glycopeptides MS/MS spectra.

Notably, to describe the Pentamer O-glycosylation pattern we used nano-LC–MS/MS approach in combination with different fragmentation methods; indeed, HCD allows to obtain information about both peptide primary sequence and glycan structure, however site-specific information is lost due to O-GalNAc linkage lability. On the other hand, Electron-Transfer Dissociation (ETD) promotes only the fragmentation of the peptide portion, maintaining O-GalNAc linkages to the protein (Wilkinson and Saldova 2020). A combination of both HCD and ETD, called EThcD, has been exploited, in order to retain all the information concerning peptide structure, glycan structure and glycosylation site (Yu et al. 2017). With this strategy three Pentamer O-glycosylation sites have been identified: S4 and T8 (on gL) and T149 (on gH). S4 brings only 1 type of glycan structure (sialyl T antigen), T8 brings three different types of glycan structures (sialyl T antigen, T antigen and Tn antigen), as well as T139 (sialyl T antigen, Core 2 and sGal Core 3). In order to discriminate between isomeric structures, the presence of characteristic oxonium ions has been checked; in fact, in the case of sT antigen, the ion at *m/z* 454.16 distinguishes it from the isomer Galβ1 → 3(Siaα2 → 6)GalNAc (Fig. S5), while in the case of sGal Core 3, the ion at *m/z* 657.24 distinguishes it from the isomer Siaα2 → 3Galβ1 → 3(GlcNAcβ1 → 6)GalNAc (Fig. S5).

**Figure 6:**
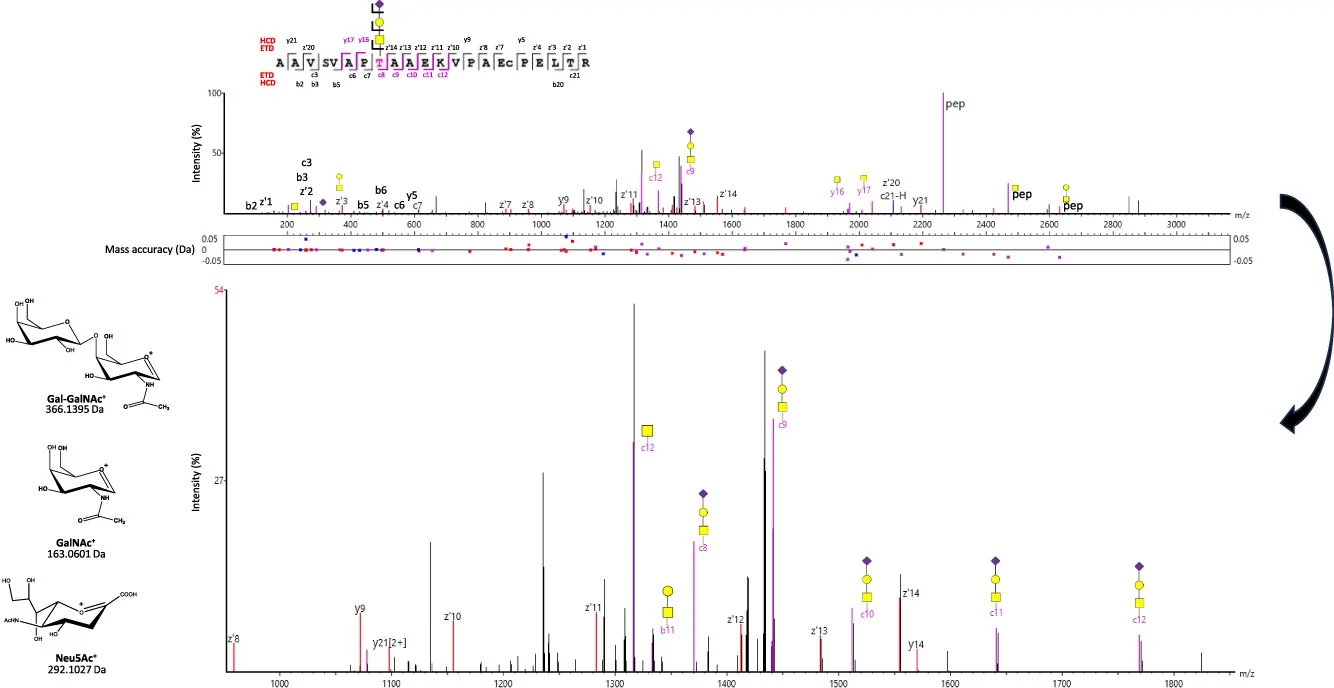
EThcD MS/MS spectrum of a tryptic O-glycopeptide harboring T8 from pentamer, with below a zoom of the region between *m/z* 950–1850 to better visualize the c-ions bringing the intact O-glycan. The ion fragments bearing monosaccharides are violet colored; the oxonium ions have been highlighted only using the corresponding SNGF structures for space reasons; cysteine carbamidomethylation is indicated with lowercase in the glycopeptide primary sequence. The structures and the mass values of the observed oxonium ions have been reported on the left of the image.

An example of detected peptide harboring O-glycosylation on the T8 is reported in Fig. 6.

**Figure 7:**
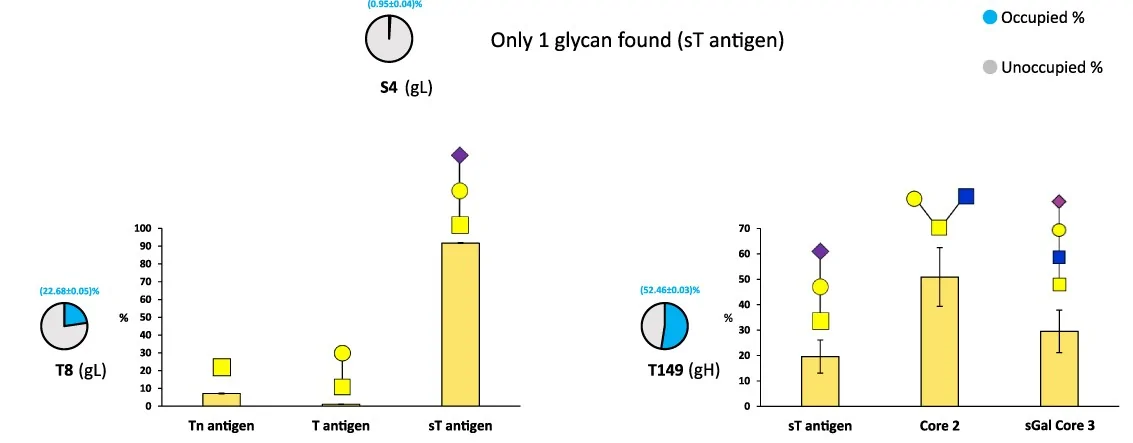
Occupancy (pie charts) and microheterogeneity (histograms) of gL and gH O-glycosylation sites from pentamer expressed in CHO cells.

Through the corresponding extracted ion chromatogram and its integration both microheterogeneity and occupancy have been assessed for each O-glycosylation site; site S4 has an occupancy of 0.95% and only one glycan structure has been identified on it. On the contrary the site T8 showed an occupancy of 22.7% and three different glycoforms, with sT antigen as being the most abundant one and site T149 showing an occupancy of 52.5% and three different glycoforms, with Core 2 oligosaccharide as the most abundant (Fig. 7).

The detected O-glycosylation sites are in agreement with those once found by Bagdonaite *et al.* on Pentamer isolated from the native cytomegalovirus (strain Towne) replicated in HEL cells (Bagdonaite et al. 2016); indeed, the construct analyzed by Bagdonaite *et al.* shares five O-glycosylation sequons with the one expressed in CHO cells (analyzed in this work). However, for the CHO cells derived Pentamer, only three sequons have been found to be unambiguously O-glycosylated; this may be due to the different conditions used to obtain the pentameric complex.

The O-glycans occupancy and microheterogeneity analysis has been performed also on Pentamer from Expi293 GnT1- cells, which gave occupancies values comparable to the ones from Pentamer expressed in CHO cells, with Pentamer from Expi293 GnT1- showing site S4 slightly more occupied and sites T8 and T149 slightly less occupied (Fig. S6); we have quite similar occupancy data because O-glycosylation happens in the Golgi apparatus when the protein is fully formed and folded, for this reason, the 3D structure of the protein plays a major role in determining which sites will be occupied and how the sugars will be processed (Rudd and Dwek 1997).

Furthermore, in the Expi293 GnT1- we have lower glycan microheterogeneity for sites T8 and T149 and higher glycan microheterogeneity for site S4 (Fig. S6).

### Pentamer immunogenicity and glycosylation pattern

To determine whether Pentamer immunogenicity is linked to the different glycosylation patterns, Pentamer was expressed in Expi293 GnT1- and CHO cells, mice were immunized and neutralizing antibodies (nAbs) titers evaluated.

The nAbs titer is expressed as geometric mean (GMT) of all the measured values (10 biological replicates), where each measurement corresponds to the dilution according to which 95% of cells are not infected (IC95) which is based on a specific starting serum dilution (the lowest dilution tested). The data have been fitted to a nonlinear regression model, which allowed us to determine the neutralizing titer elicited by each antigen at 95% confidence interval (CI).

**Figure 8:**
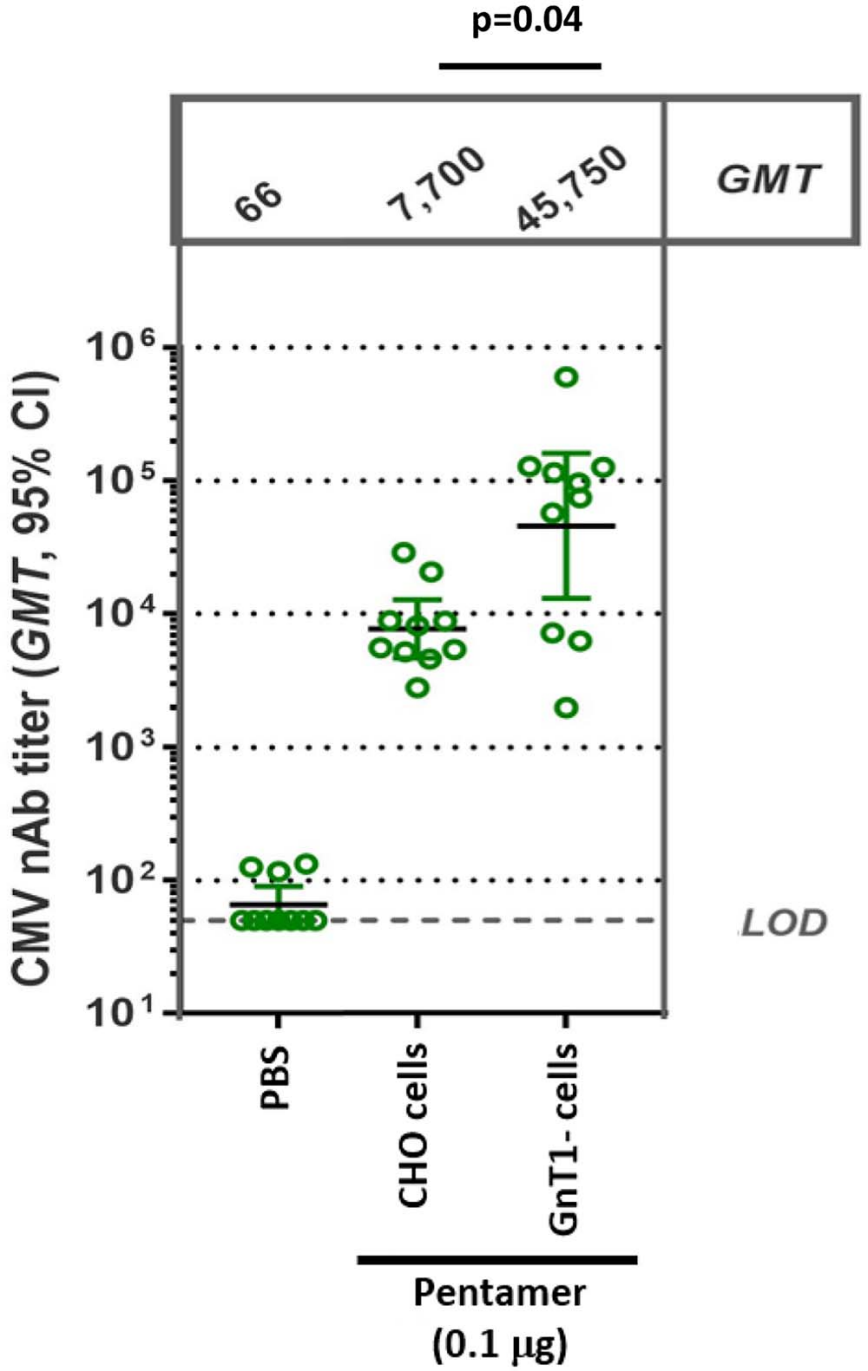
Immunogenicity of pentamer (from CHO cells and Expi293GnT1- cells). GMT = geometric mean. LOD = limit of detection. The p-value of the *t-*test with level of significance (p) of 0.04 is reported.

**Figure 9:**
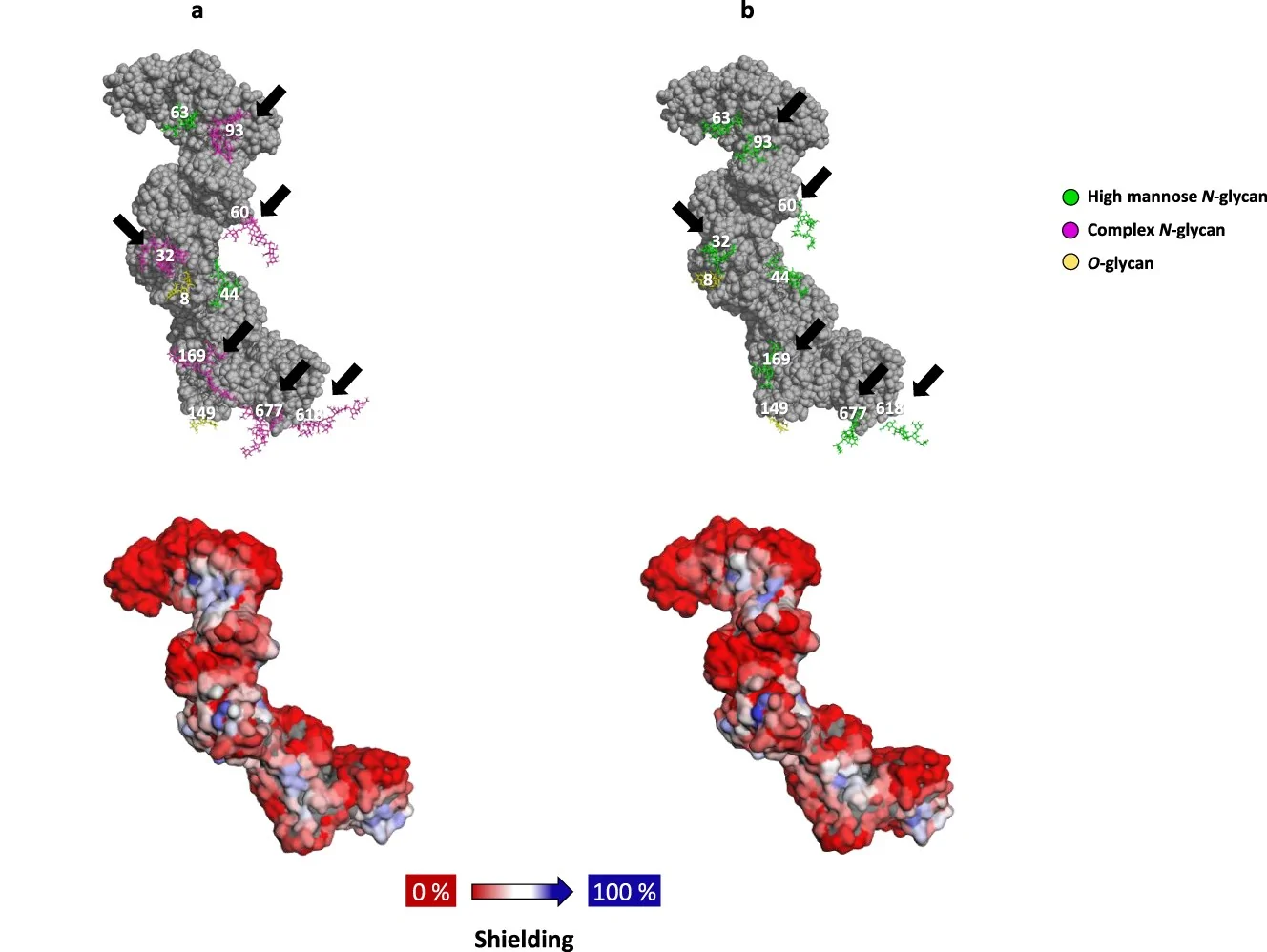
The pentamer protein backbone atoms are shown as gray dot spheres and the glycans are shown as stick models of different colors depending on the most abundant glycoform at that site. a) Pentamer from CHO cells. b) Pentamer from Expi293 GnTI- cells. Glycans with diverse bulkiness between the two structures are indicated by the arrows. Sites N39 (gH), and N44 (gH) have not been reported, S4 (gL) is not present in the crystal (9 out 11 N-glycosylation sites and 2 out 3 O-glycosylation sites are visualized in the crystal). The glycosylated model was constructed using the publicly available 3D structure (PDB: 5VOD) and GlycoSHIELD (Tsai et al. 2024). The corresponding 3D heatmaps of the accessible surface area (ASA) of the two pentamer forms are reported below (computed by GlycoSHIELD). In grey are represented the intrinsically inaccessible protein regions.

Pentamer expressed in Expi293 GnT1- and harboring only oligomannose glycosylation elicited a higher titer of nAbs (about six folds) than Pentamer expressed in CHO cells with a complex glycosylation decoration (Fig. 8). Pentamer from Expi293 GnT1- cells is decorated with only M5 glycans, which play a lower steric hindrance than Pentamer from CHO cells, boosting in this way the antigen immunogenicity; indeed, it is possible to visualize from the tridimensional model of Pentamer from Expi293GnTI- cells how there is a higher portion of protein backbone exposed (Fig. 9). Furthermore, epitope shielding has been quantified by GlycoSHIELD, using a tridimensional heatmap of the accessible surface area (ASA), which confirms how regions next to the epitopes for neutralizing antibodies, 2C12, 8I21, MSL-109 are less shielded in the Pentamer from Expi293 GnTI- cells (Fig. 9), (Ciferri et al. 2015b; Chandramouli et al. 2017; Kschonsak et al. 2022; Tsai et al. 2024). Furthermore, glycans on N60, N63, N93 are involved in the shielding of two neutralizing epitopes on the tip of the complex (mAbs 2C12, 8I21), while glycan on N169 is involved in the shielding of the neutralizing epitope of the mAb MSL-109 at the base of the glycoprotein (Fig. S7A). Glycan can hamper the recognition of protein epitopes by B-cells immunoglobulins, lowering the elicitation of neutralizing antibodies after vaccine administration; this happens because oligosaccharides are highly conformationally mobile structures, which sweep a wide space region around a specific protein region, creating a shield which prevents interactions between the protein backbone and external molecules (Fig. S7B).

## Discussion

Structure, abundance and occupancy of the glycans decorating HCMV Pentamer in different cell lines have been unrevealed exploiting high resolution mass spectrometry; afterwards, the impact of the different glycosylation patterns on antigen immunogenicity has been assessed by neutralization assay.

N-glycans site-specific analysis has been performed on Pentamer from CHO cells; the Pentameric complex showed all the N-glycosylation sites to be 100% occupied, with only two of them bearing mainly high-mannose structures. All these data suggest that there are protein regions less accessible to processing enzymes than others, probably due to glycan structures that are sandwiched between other two. The Pentameric complex has all the N-glycosylation sites occupied, with only two over nine sites analyzed bearing mainly high-mannose structures. Glycan maturation has been discussed considering the steric effect of the assembled Pentamer (and not of the single subunits) because the assembly of multimeric complexes is required to pass the ER quality control checkpoint that precedes transport through the Golgi apparatus, where oligosaccharides maturation starts. (Ellgaard et al. 1999), (Moremen and Maurizio 2006).

N-glycans profiling experiments have been performed on Pentamer from CHO cells and Pentamer from GnT1-cells. The different expression conditions showed a totally different N-glycans profile between the two glycoproteins, with Pentamer from GnT1- having simpler profiles with only few high-mannose structures. On the other hand, Pentamer from CHO cells showed crowded profiles due to the presence of several complex glycans structures.

Pentamer O-glycosylation pattern has been studied using tandem mass spectrometry with different fragmentation methods: HCD, ETD and EThcD. A boost in sensitivity for this kind of analysis has been obtained by using a nano-LC system. The analysis brought to the identification of three O-glycosylation sites (Ser4, Thr8 and Thr149) and five different glycoforms (sialyl T antigen, T antigen and Tn antigen, Core 2 and sGal Core 3). The ability to elicit the production of neutralizing antibodies in mice has been studied both for Pentamer with different glycosylation patterns; specifically, the immunogenicity of Pentamer from CHO cells and Pentamer from Expi293 GnT1- cells have been compared. Pentamer from Expi293GnTI- cells showed higher immune response than Pentamer from CHO cells. The effect of the glycan shield on the protein epitopes has been assessed by building 3D models and SASA heatmaps of the antigens exploiting the software GlycoSHIELD (Tsai et al. 2024). Pentamer from CHO cells showed a higher shielding of the immunogenic epitopes due to the presence of bulkier complex glycans, on the opposite, Pentamer from Expi293 GnT1- cells showed a lower shielding of the immunogenic epitopes thanks to the shorter M5 and M6 oligosaccharides.

## Materials and methods

### Expression of HCMV pentamer in CHO cells

CHO cells (CHO-K1-C8TD cell line) have been co-transfected with separate plasmids expressing the individual components gH, gL, UL128, UL130, UL131A. Recombinant HCMV Pentamer (Merlin strain) with a truncation of the cytoplasmic and transmembrane domains of gH was expressed in CHO cells (CHO-K1-C8TD cell line), grown in CD-CHO medium (Invitrogen, Carlsbad, CA, USA), in the presence of 5% of O_2_; pH was controlled at the set point of 7.00 with a dead band of 0.05 and controlled either via addition of 1.0 M NaOH or by sparging CO_2_ as needed, with an agitation set at 100 rpm. Starting day 2, a defined amount of ADCF Antifoam, irradiated (3% Hyclone (Foamaway™)) was regularly added. Temperature was controlled at 37 °C during the first 2 days and then shifted to 33 °C to initiate the production phase, which lasts 12 days. After the production phase, the harvest containing the secreted glycoprotein was clarified using a series of filters (0.5/0.2 μm), then the antigen was purified with a Nuvia C-prime resin (Bio-RAD) in bind/elute mode; virus inactivation was then performed by adjusting the pool to a target concentration of 2% (*v/v*) Polysorbate80 and stored overnight at room temperature after a 0.22 μm filtration. The virus-inactivated pool was diluted and loaded onto an anion exchange chromatography column with HP-Q resin (Bio-RAD) in bind/elute mode. The HP-Q pool was adjusted in conductivity and loaded onto on a multimodal chromatography column with CHT type I resin (Bio-Rad) in flowthrough mode. At the end of the chromatographic purification the pool undergoes a series of filtrations.

### Expression of HCMV pentamer in Expi293 GnT1- cells

Expi293 GnT1- cells have been co-transfected with separate plasmids expressing the individual components gH, gL, UL128, UL130, UL131A. HCMV Pentamer (Merlin strain), with a truncation of the cytoplasmic and transmembrane domains of gH, was stably expressed in human embryonic kidney (HEK) 293S cells bringing GnT1- mutation. The harvested extracellular broth was loaded directly onto a StrepTrap HP column (GE Healthcare), and the proteins were eluted with 2.5 mM desthiobiotin (IBA Lifesciences). The Strep tag was cleaved off by incubating TEV protease (AcTEV, Thermo Fisher Scientific) overnight at 4 °C. The sample was diluted with 20 mM HEPES buffer (pH 7.0) to lower total salt concentration to 50 mM and loaded onto Mono S 10/30 column (GE Healthcare). Pentamer was eluted on a salt gradient from 50 mM to 1.0 M NaCl.

### SDS-page

For sodium dodecylsulfate-polyacrylamide gel electrophoresis (SDS-PAGE) analysis, 10 μg (protein content) of Pentamer samples were solubilized in LDS sample buffer (Thermo Fisher Scientific) with 10 mm DTT, boiled for 5 min, separated on NuPAGE™ Novex™ 4%–12% polyacrylamide Bis-Tris protein gel (Thermo Fisher Scientific) and stained with Coomassie Blue R-250. SeeBlue Plus2 Pre-Stained Protein Standard has been used and molecular weight ladder.

### Size exclusion chromatography analysis

50 μg of Pentamer from CHO and Expi293 GnT1- cells have been analyzed by SEC-UV system. The analysis was performed on an ACQUITY UPLC I-Class (Waters) coupled to an ACQUITY UV detector. Proteins were separated using a TSKgel 3000SWxl, (250 Å, 5 μm, 0.78 × 30 cm, Tosoh Bioscience), the mobile phase was 10 mM KH_2_PO_4_ 300 mM (NH_4_)_2_SO_4_, pH 7.3, the UV detector was operated with an excitation wavelength of 214 nm.

### LC–MS/MS analysis experimental details for N-glycosylation

Protein denaturation and reduction was done by diluting 28 μg of Pentamer from Expi293 GnT1- cells with 82 μL of 8 M guanidinium chloride, adding 1.5 μL of 1 M DTT (with final concentration of 15 mM) and incubating for 30 min at 50 °C; then the free cysteines were alkylated by adding 7.5 μL iodoacetamide to obtain a 30 mM solution, the sample was incubated for 30 min at room temperature in absence of light. The remaining iodoacetamide was quenched by adding 3 μL of 1 M DTT to obtain a 30 mM solution. The sample was buffer exchanged with 50 mM ammonium bicarbonate buffer pH = 7.8.

The glycoprotein was digested overnight at 37 °C with a proteolytic enzyme (trypsin, chymotrypsin or α-lytic protease) using an enzyme:substrate ratio of 1:20.

Glycopeptides were purified before LC–MS/MS analysis by SPE with OASIS HLB Extraction Cartridges 1 cc. The elution volume was then split into two halves. The first half was dried, dissolved in 100 μL of 0.1% (v/v) formic acid (HCOOH) in H_2_O, centrifuged and injected (5 μL) onto LC–MS/MS system form site-specific glycans microheterogeneity analysis.

The second half of the volume was dried and dissolved in 50 mM ammonium bicarbonate in H_2_^18^O and 2 μL of PNGase F (500 units/mL, in H_2_^18^O) were added to the sample allowing an incubation at 37 °C for 1 h.

The sample then was acidified with 10% (v/v) HCOOH and injected at the instrument form site-specific glycans occupancy analysis; each sample was prepared in triplicate and each injection was repeated three times.

The analyses were performed on an ACQUITY UPLC I-Class (Waters) to a Q-Exactive Plus Mass Spectrometer (Thermo Scientific). Peptides and glycopeptides were separated using an Acquity UPLC Peptide CSH C18, (130 Å, 1.7 μm, 1 × 150 mm, Waters) the mobile phases were 3% (v/v) acetonitrile, 0.1% (v/v) formic acid in water (phase A) and 0.1% (v/v) formic acid in acetonitrile (phase B), the column temperature was kept at 60 °C. The Q-Exactive Plus Mass Spectrometer was operated in Full MS/data dependent MS^2^ mode (Full MS/dd-MS^2^), 3 × 10^6^ ions were accumulated in the Orbitrap (Automatic Gain Control, AGC), the mass range used was *m/z* 300–3500; dynamic exclusion was enabled for 12 s, scans were acquired at 70,000 mass resolving power (full MS), normalized Collision Energy (NCE) of 26 was used.

The electrospray ion source temperature was set at 320 °C and the capillary voltage was set to 3.4 kV. Xcalibur was used for chromatogram integration and quantitative analysis. PEAKS Studio 11 was used to perform the peptide mapping experiment and to attribute the glycoforms to each *N*-glycosylation site. A false discovery rate (FDR) of 1% was used to detect glycopeptides; the precursor ion mass error tolerance was of 15 ppm, the fragment mass error tolerance was of 0.05 Da; moreover, to correctly describe a glycopeptide the following criteria have been used for manual inspection: the glycopeptide must be characterized by at least two MS/ MS spectra with peptide and glycan scores (-10LogP_value_) of 15 at least for each portion, mass accuracy of the precursor ion of maximum 5 ppm, and each MS/MS spectrum must contain at least two oxonium ions from glycan fragmentation, which have been used also to verify correct glycan identification. The glycoforms abundances were calculated by integration of the MS1 chromatogram, using the software PEAKS Studio 11.

### Enzymatic release and fluorophore labeling of N-glycans

15 μg of Pentamer from CHO cells or Expi293 GnT1- cells were dispensed in two different 1.5 mL Eppendorf. Protein denaturation was done by adding 6 μL of 5% (w/v) RapiGest and 23 μL of water, then the sample it has been heat denatured for 5 min at 90 °C. To remove *N*-glycans from protein, 1.2 μL of Rapid PNGase F have been added and the sample has been incubated at 50 °C for 10 min. For *N*-glycans labelling, 12 μL of 7% (w/v) RapiFluor solution (in dimethylformamide) have been added to the test tube and it has been incubated 5 min at room temperature. Protein has been precipitated by adding 50 μL dimethylformamide/acetonitrile (32%/68%). This procedure was repeated 2 times, in order to obtain 2 technical replicates.

The analyses were performed on an ACQUITY UPLC I-Class (Waters) coupled to an ACQUITY Premier fluorescence (FLR) detector and to a Q-Exactive Plus Mass Spectrometer (Thermo Scientific). Labelled N-glycans were separated using an Acquity UPLC Glycan BEH Amide, (130 Å, 1.7 μm, 2.1 × 150 mm, Waters) the mobile phases were 50 mM ammonium formate, pH 4.4 (phase A) and acetonitrile (phase B), the column temperature was kept at 60 °C.

The FLR detector was operated with excitation wavelength of 265 nm and emission wavelength of 425 nm; the FLR data scan rate was 2 points/sec. The Q-Exactive Plus Mass Spectrometer was operated in Full MS/data dependent MS^2^ mode (Full MS/dd-MS^2^), 3 × 10^6^ ions were accumulated in the Orbitrap (Automatic Gain Control, AGC), the mass range used was *m/z* 300–3500; dynamic exclusion was enabled for 12 s, scans were acquired at 70,000 mass resolving power (full MS), normalized Collision Energy (NCE) of 26 was used.

The electrospray ion source temperature was set at 320 °C and the capillary voltage was set to 3.4 kV. Chromeleon was used for chromatogram integration and quantitative analysis.

### O-glycosylation analysis

Protein denaturation and reduction was done by diluting 28 μg of Pentamer from CHO-K1-C8TD cells or Expi293 GnT1- cells with 82 μL of 8 M guanidinium chloride, adding 1.5 μL of 1 M DTT (with final concentration of 15 mM) and incubating for 30 min at 50 °C; then the free cysteines were alkylated by adding 7.5 μL of iodoacetamide to obtain a 30 mM solution, the sample was incubated for 30 min at room temperature in absence of light. The remaining iodoacetamide was quenched by adding 3 μL of 1 M DTT to obtain a 30 mM solution. The sample was buffer exchanged with 50 mM ammonium bicarbonate buffer pH = 7.8.

The glycoprotein was digested overnight at 37 °C with a proteolytic enzyme (trypsin or chymotrypsin) using an enzyme:substrate ratio of 1:20.

Glycopeptides were purified before nLC-MS/MS analysis by SPE with OASIS HLB Extraction Cartridges 1 cc. Then the sample was dried, dissolved in 50 μL of 0.1% HCOOH, centrifuged and injected (5 μL) at nLC-MS/MS system form site-specific O-glycans analysis.

The analyses were performed on a Vanquish Neo UHPLC (Thermo Scientific) coupled to an Orbitrap Eclipse Tribrid Mass Spectrometer (Thermo Scientific). Peptides and glycopeptides were separated using an μPAC™ Neo HPLC Column, (20 μm ×50 mm, Thermo Scientific) the mobile phases were 3% (v/v) DMSO, 0.1% (v/v) formic acid in water (phase A) and 3% (v/v) DMSO, 0.1% (v/v) formic acid, 80% (v/v) acetonitrile (phase B).

The Orbitrap Eclipse Tribrid Mass Spectrometer was operated in Full MS/data dependent MS^2^ mode (Full MS/dd-MS^2^), a 400,000 AGC target has been used, the mass range used was *m/z* 350–1200; dynamic exclusion was enabled for 30 s, scans were acquired at 120,000 mass resolving power (full MS), EThcD was the activation type used, a supplemental activation (25%) of the charge-reduced species was used in the ETD analysis to improve fragmentation.

The electrospray ion source temperature was set at 275 °C and the capillary voltage was set to 1.9 kV. Xcalibur was used for chromatogram integration and quantitative analysis. PEAKS Studio 11 was used to perform the peptide mapping experiment and to attribute the glycoforms to each N-glycosylation site. A false discovery rate (FDR) of 1% was used to detect glycopeptides; the precursor ion mass error tolerance was of 15 ppm, the fragment mass error tolerance was of 0.05 Da. The glycoforms abundances were calculated by integration of the MS1 chromatogram, using the software PEAKS Studio 11.

### nAbs titer sample preparation and analysis

Animal studies were ethically reviewed and carried out in compliance with EU DIR 63/2010. For each antigen, immunization has been performed on ten female laboratory mice, strain C57BL/6, at 7–10 weeks of age; 0.15 mg of Pentamer were administered by intramuscular vaccinations with 50 μL/animal in 1 hind leg (quadriceps). The mice have been vaccinated every three weeks, for a total of three immunizations; blood samples were collected at day 21, 42 and 64 after first immunization. Serum was collected after blood centrifugation (10,000 × *g*) for 10 min at room temperature.

To measure the nAbs titer, virus solution was preincubated with serial dilutions (1:1000, 1:2000, 1:4000, 1:8000, 1:16000, 1:32000, 1:64000, 1:128000) of immunized mouse serum in 37 °C, 5% CO_2_ incubator for 2 h. Then, 50 μL of the virus/serum mixture were added to the wells of the assay plate containing a layer of ARPE-19 cells and they incubated overnight at 37 °C, 5% CO_2_ incubator. Therefore, the cells have been washed with PBS and fixed with 50 μL of 4% (w/v) paraformaldehyde at room temperature for 20 min. Cells have been washed once again with PBS and 50 μL of 0.1% (w/v) Triton X-100 have been added to the wells to permeabilize cells. After washing with PBS, 50 μL of primary antibody have been added to the plate (mouse anti-CMV IE1 monoclonal antibody); the system has been incubated in 37 °C incubator for 1 h. Then, the cells have been washed with PBS and 50 μL of secondary antibody (Alexa Fluor mouse anti-IgG) and 4′,6-diamidino-2-phenylindole (DAPI), a fluorescent substrate, have been added; the system has been incubated in 37 °C incubator for 1 h. After PBS washing with PBS, the plates have been read using the High Content Imaging-CX7. To verify if the difference between the immune responses of two groups (CHO cells and Expi293GnT1- cells) is statistically significant, independent two-sample *t-*test has been carried out, using a threshold for statistical significance (α) of 0.05; the p-value showed to be of 0.04, so p < α, proving that the two immune responses are statistically different.

### Model construction

Pentamer faces (Fig. 1c) have been decorated with 30 N-glycan conformers for each N-glycosylation site using GlycoSHIELD. The following parameters have been used for the modelling: grafting mode CG, grafting cutoff of 3.0 and every 10^th^ glycan conformer has been considered for grafting.

Pentamer (Fig. S7B) has been decorated with 30 N-glycan conformers for each N-glycosylation site using GlycoSHIELD. The following parameters have been used for the modelling: grafting mode CG, grafting cutoff of 3.0 and every 10^th^ glycan conformer has been considered for grafting.

Structural models of N-linked glycan presentation on Pentamer (Fig. 3) were created using the Pentamer crystal structure (PDB ID code 5VOD) and GlycoSHIELD with the same parameters reported above.

Heatmap of the SASA (Fig. 9) was calculated using the Pentamer crystal structure (PDB ID code 5VOD) and the tool software GlycoSHIELD, by performing the simulation on 30 N-glycan conformers for each N-glycosylation site and setting the probe diameter to 0.75 nm (size of an antibody complementarity-determining region) and the number of dots per sphere to 15.

## Supplementary Material

Supplementary_materials_cwag062

## Data Availability

Data will be made available on request.

## Fundings

This work was supported by GlaxoSmithKline Biologicals SA; AL was a recipient of a PhD grant sponsored by GlaxoSmithKline Biologicals SA.
