# Supplementary material for: Site-specific and global glycan analysis of the human cytomegalovirus pentameric glycoprotein by high resolution mass spectrometry
Source: Glycobiology. 2026 Aug 5;36(10):cwag062. doi: 10.1093/glycob/cwag062 (PMC13513752; doi:10.1093/glycob/cwag062)
Supplement: Supplementary file 1 [file supplementary_materials_cwag062.zip › Supplementary materials_unmarked.docx]

**Supplementary Information**

**Site-specific and global glycan analysis of the human cytomegalovirus pentameric glycoprotein by high resolution mass spectrometry**

**Authors**

Antonio Lembo^a,b^, Gian Luca Sardone^b^, Michela Aurilia^b^, Immacolata Speciale^a^, Cristina De Castro^a^, Massimiliano Biagini^b^

*^a^Department of Chemical Sciences, University of Naples Federico II, Naples, Italy*

*^b^GSK Vaccines, Analytical R&D, Siena, Italy*

# **Supplementary table 1.** Site specific *N*- and *O*-glycans microheterogeneity analysis from Pentamer (CHO cells) analysis data

|  | N32 (gH. chymotrypsin) |  |
| --- | --- | --- |
| Glycan | Relative abundance (%) | Standard deviation |
| N3F G1F **S2F** | 5.94  10.29  **83.77** | 1.71  5.36  **3.66** |
|  | | |

|  | N169 (gH. α-lytic protease) |  | |
| --- | --- | --- | --- |
| Glycan | Relative abundance (%) | Standard deviation | |
| G0F G1F G2F S1G1F G2N3F **S1G2F** S2F G1N3F S1G3F | 0.69  3.90  8.69  6.01  0.81  **74.86**  3.78  0.80  0.46 | 0.15  1.65  0.78  2.60  0.18  **6.71**  1.51  0.30  0.15 |  |
|  | | | |

|  | N618 (gH. chymotrypsin) |  |
| --- | --- | --- |
| Glycan | Relative abundance (%) | Standard deviation |
| S2G3F  S2F  S2G1  **S1G4** | 8.34  12.13  11.54  **67.99** | 2.82  3.01  1.72  **7.54** |
|  |  |  |

|  | N677 (gH. trypsin) |  |
| --- | --- | --- |
| Glycan | Relative abundance (%) | Standard deviation |
| M6 M5 M4 G0M4 G0M5 S1M3 S1M4 S1M5 G0 G1 G2 S1G1 S2G0 **S1G2** S2G1 S2 S2F | 0.65  1.14  0.36  0.75  0.50  2.58  2.36  1.92  0.73  2.36  4.24  4.56  1.32  **32.16**  30.33  8.18  5.85 | 0.14  0.19  0.11  0.15  0.19  0.06  0.07  0.80  0.09  0.26  0.34  0.20  0.60  **1.39**  1.20  0.61  0.75 |
|  | | |

|  | N44 (gL. chymotrypsin) |  |
| --- | --- | --- |
| Glycan | Relative abundance (%) | Standard deviation |
| **M5**  M6  M7  G0M5  S1M3  G1M5  S1M4  S1M5  G2  S1G2  S1G2F | **32.11**  16.50  7.84  6.49  3.17  7.67  10.33  6.26  1.57  5.71  2.36 | **3.84**  1.20  0.96  1.07  0.29  0.67  1.04  2.48  0.27  2.33  0.97 |
|  |  |  |

|  | N63 (UL131A. trypsin) |  |
| --- | --- | --- |
| Glycan | Relative abundance (%) | Standard deviation |
| M7  M6  M5  M4  S1M5  G0M5  S1M4  G1M5  G0M7  G0  G2 | 23.10  46.21  21.11  0.30  2.89  0.65  0.39  5.13  0.07  0.10  0.05 | 1.15  0.77  0.71  0.06  0.19  0.10  0.03  0.47  0.00  0.01  0.03 |

|  | N60 (UL130. trypsin) |  |
| --- | --- | --- |
| Glycan  G0M3F  N3F  G0F  G1F  G2F  G3F  G2N3F  G1N3F  S1G1F  S1G2F  S2G1F  S2G3  S2G3F | Relative abundance (%)  0.12  0.73  1.50  4.25  24.81  1.37  0.41  1.59  3.08  52.09  0.64  2.95  6.46 | Standard deviation  0.05  0.02  0.10  0.55  3.92  0.67  0.11  0.26  0.89  2.78  0.04  0.59  2.16 |

|  | N93 (UL130. trypsin) |  |
| --- | --- | --- |
| Glycan  G0M3  G0  G1  G1F  G2  S1G1  G2F  S1G2  S1G2F | Relative abundance (%)  0.49  1.65  2.47  0.45  14.19  7.29  4.71  42.67  26.06 | Standard deviation  0.18  0.84  0.15  0.19  1.40  0.74  2.15  8.94  8.47 |

|  | N176 (UL130. trypsin) |  |
| --- | --- | --- |
| Glycan  M5  G1  S2G0  S2 | Relative abundance (%)  0.37  72.92  23.94  2.77 | Standard deviation  0.20  2.57  1.08  1.41 |

|  | S4 (gL. trypsin) |  |
| --- | --- | --- |
| Glycan  sT antigen | Relative abundance (%)  100 | Standard deviation  n.a |

|  | t8 (gL . trypsin) |  |
| --- | --- | --- |
| Glycan  Tn antigen  T antigen  sT antigen | Relative abundance (%)  7.12  1.12  91.75 | Standard deviation  0.10  0.15  0.10 |

|  | t149 (gH. chymotrypsin) |  |
| --- | --- | --- |
| Glycan  sT antigen  Core 2  GlcNAc sT | Relative abundance (%)  19.59  50.91  29.50 | Standard deviation  5.00  10.00  6.00 |

The most abundant glycan for each site is highlighted in bold.

# **Supplementary table 2.** Site specific *N*- and *O*-glycans occupancy analysis from Pentamer (CHO cells) analysis data

|  | gH |  |
| --- | --- | --- |
| Asn | Occupancy (%) | Standard deviation |
| N32 | 100 | n.a. |
| N39  N44  N169  N618  N677  t149 | 95.45  100  99.88  100  96.60  52.46 | 0.02  n.a.  0.02  n.a.  0.29  0.03 |

|  | gL |  |
| --- | --- | --- |
| Asn | Occupancy (%) | Standard deviation |
| N44 | 100 | n.a. |
| S4  t8 | 0.95  22.68 | 0.04  0.05 |

|  | ul130 |  |
| --- | --- | --- |
| Asn | Occupancy (%) | Standard deviation |
| N60  N93 | 100  99.50 | n.a.  0.04 |
| N176 | 99.27 | 0.03 |

|  | ul131a |  |
| --- | --- | --- |
| Asn | Occupancy (%) | Standard deviation |
| N63 | 98.94 | 0.01 |
|  |  |  |

# **Supplementary table 3.** Released N-glycans from Pentamer_GnT1-_ analysis data

| Peak number | Retention time (min) | Glycan | Experimental  mass (Da) | Theoretical accurate mass (Da) | Accuracy  (Δppm) | Relative abundance (%) | Standard deviation |
| --- | --- | --- | --- | --- | --- | --- | --- |
| 1  2  3  4  5 | 12.2  15.4  16.5  18.3  20.0-20.9 | M4  M5  M5F  M6  M7 | 1384.5630  1546.6158  1692.6737  1708.6687  1870.7215 | 1384.56628  1546.61743  1692.67761  1708.67371  1870.72681 | 2.37  1.05  2.31  2.93  2.84 | 1.73  90.18  2.35  4.78  3.28 | 0.037  0.030  0.244  0.049  0.085 |
|  |  |  |  |  |  |  |  |

**Supplementary table 4.** Released N-glycans from Pentamer_CHO_ analysis data

| Peak number | | Retention time (min) | Glycan | Experimental  mass (Da) | Theoretical accurate mass  (Da) | Accuracy  (Δppm) | Relative abundance (%) | Standard deviation |
| --- | --- | --- | --- | --- | --- | --- | --- | --- |
| 1  2  3  4  5  6  7  8  9  10  11  12  13  14  15  16  17  18  19  20  21  22  23  24  25  26  27 | 11.35  13.06  16.83  15.48  15.93  16.23  16.88  17.25  17.49  18.27/18.40  18.27  19.07  19.59  20.33  20.75  20.90  21.52  21.77  20.86  20.73  23.04  23.45  23.98  24.95  26.35  27.08  27.81 | | G0M3  G0  G0M5  M5  G1  S1M3  G1F  S1M3F  S1G1  G2+S1M4  M6  G2F  S1G2  S1G2F  S1M5  M7  S2F  S1'G2F  S2  G3F  S2F2  S1G3F  S2G3F  S3F  S2G4F  S3G4F  S4G4F | 1425.5896  1628.6689  1749.6952  1546.6158  1790.7218  1878.7378  1936.7797  2024.7957  2081.8172  1952.7746/2040.7906  1708.6687  2098.8247  2243.8700  2389.9279  2202.8357  1870.7215  2681.0233  2405.9229  2534.9655  2202.8435  2827.0813  2755.0601  3046.1556  3337.2510  3411.2877  3702.3832  3993.4786 | 1425.5917  1628.6692  1749.7007  1546.6191  1790.7281  1878.7433  1936.7849  2024.8082  2081.8225  1952.7737/2040.7947  1708.6717  2098.8342  2243.8787  2389.9392  2202.8447  1870.7268  2681.0303  2405.9362  2534.9792  2202.8494  2827.0952  2755.0742  3046.1704  3337.2693  3411.2949  3702.3923  3993.4866 | 1.47  0.18  3.14  2.13  3.52  2.93  2.68  6.17  2.55  -0.46/2.01  176  4.53  3.88  4.73  4.09  2.83  2.61  5.53  5.40  2.68  4.92  5.12  4.86  5.48  2.11  2.46  2.00 | 0.10  0.47  0.15  4.16  0.37  0.31  2.41  0.49  1.22  0.36  10.90  4.93  9.07  29.71  1.70  8.62  12.17  0.52  1.42  0.56  1.26  1.22  3.39  2.78  0.47  0.52  0.71 | 0.001  0.31  0.047  0.021  0.009  0.003  0.012  0.005  0.003  0.001  0.007  0.044  0.083  0.10  0.068  0.009  0.071  0.022  0.010  0.021  0.038  0.019  0.043  0.006  0.025  0.019  0.014 |
|  |  | |  |  |  |  |  |  |

**Figure S1.** Annotated fragmentation mass spectra of N677 gH glycopeptides, each one containing the same N-glycosylation sequon and bringing different glycan types. Pink-coloured signals are attributed to either peptide fragment ions bringing a saccharide portion or glycan oxonium ions. S2G1 and S2G0 glycans oxonium ions masses are specified.

**Figure S2.** List of all the glycan structures found on the analyzed glycoproteins.

**Figure S3.** Annotated fragmentation mass spectra of glycopeptides after treatment with PNGase F in H_2_^18^O. Asparagines that owed a glycan are indicated with lowercase N.

**Figure S4.** Fragmentation mass spectra of N-glycans labeled with Rapifluor-MS.

**Figure S5.** Oxonium ions region of EThcD spectra of O-glycopeptides bringing sT antigen and sGal Core 3 structure.

**Figure S6.** Occupancy (pie charts) and microheterogeneity (histograms) of gL and gH O-glycosylation sites from Pentamer expressed in Expi293 GnT1- cells.

**Figure S7.** A) Pentamer neutralizing epitopes affected by glycan shielding. 2C12 is a linear epitope, while 8I21 and MSL-109 are conformational epitopes. B) Conformational mobility of M5 N-glycans on Pentamer; red colored conformers play a more localized shielding effect of the protein backbone due to their lower conformational freedom.
